# Supplementary material for: H3K36 methylation and DNA-binding both promote Ioc4 recruitment and Isw1b remodeler function
Source: Nucleic Acids Res. 2022 Feb 21;50(5):2549–65. doi: 10.1093/nar/gkac077 (PMC8934638; doi:10.1093/nar/gkac077)
Supplement: gkac077_Supplemental_File [file gkac077_supplemental_file.pdf]

H3K36 methylation and DNA-binding both promote Ioc4 recruitment and Isw1b remodeler function

Jian Li [1,2], Lena Bergmann [1,3], Andreia Rafael de Almeida [3], Kimberly M. Webb [4], Madelaine M. Gogol [5], Philipp Voigt [4], Yingfang Liu[2,6], Huanhuan Liang [2,7]* and Michaela M. Smolle [3,8]*

Supporting Information

Strain	Parental	Genotype	Source
BY4741	BY4741	*MATa his3Δ1 leu2Δ0 met15Δ0 ura3Δ0*	Open Biosystems
YMS061	BY4741	*MATa his3Δ1 leu2Δ0 met15Δ0 ura3Δ0 IOC4-TAP::HIS3*	Open Biosystems
YMS087	BY4741	*MATa his3Δ1 leu2Δ0 met15Δ0 ura3Δ0 IOC4-3xFlag::loxP set2Δ::LEU2*	M. Smolle (1)
YMS205	BY4741	*MATa his3Δ1 leu2Δ0 met15Δ0 ura3Δ0 ioc4Δ::HIS3 chd1Δ::KanMX*	This study
YMS254	BY4741	*MATa his3Δ1 leu2Δ0 met15Δ0 ura3Δ0 IOC4-3xFlag::HIS3*	This study
YMS255	BY4741	*MATa his3Δ1 leu2Δ0 met15Δ0 ura3Δ0 IOC4*$_{\Delta 43\text{-}105}$*-3xFlag::HIS3*	This study
YMS262	BY4741	*MATa his3Δ1 leu2Δ0 met15Δ0 ura3Δ0 IOC4-3xFlag::HIS3 chd1Δ::HphB*	This study
YMS263	BY4741	*MATa his3Δ1 leu2Δ0 met15Δ0 ura3Δ0 IOC4*$_{\Delta 43\text{-}105}$*-3xFlag::HIS3 chd1Δ::HphB*	This study
YMS264	BY4741	*MATa his3Δ1 leu2Δ0 met15Δ0 ura3Δ0 IOC4*$_{\Delta 1\text{-}178}$*-3xFlag::HIS3*	This study
YMS265	BY4741	*MATa his3Δ1 leu2Δ0 met15Δ0 ura3Δ0 IOC4*$_{K149E\ K150E}$*-3xFlag::HIS3*	This study
YMS266	BY4741	*MATa his3Δ1 leu2Δ0 met15Δ0 ura3Δ0 IOC4*$_{\Delta 1\text{-}178}$*-3xFlag::HIS3 chd1Δ::HphB*	This study
YMS267	BY4741	*MATa his3Δ1 leu2Δ0 met15Δ0 ura3Δ0 IOC4*$_{K149E\ K150E}$*-3xFlag::HIS3 chd1Δ::HphB*	This study
YMS333	BY4741	*MATa his3Δ1 leu2Δ0 met15Δ0 ura3Δ0 IOC4*$_{\Delta 43\text{-}105}$ *-TAP::URA3*	This study
YMS334	BY4741	*MATa his3Δ1 leu2Δ0 met15Δ0 ura3Δ0 IOC4*$_{\Delta 1\text{-}178}$ *-TAP::URA3*	This study
YMS335	BY4741	*MATa his3Δ1 leu2Δ0 met15Δ0 ura3Δ0 IOC4*$_{K149E\ K150E}$ *-TAP::URA3*	This study
YMS376	BY4741	*MATa his3Δ1 leu2Δ0 met15Δ0 ura3Δ0 IOC4*$_{W22A}$*-3xFlag::HIS3*	This study
YMS378	BY4741	*MATa his3Δ1 leu2Δ0 met15Δ0 ura3Δ0 IOC4*$_{W22A}$*-TAP::URA3*	This study
YMS379	BY4741	*MATa his3Δ1 leu2Δ0 met15Δ0 ura3Δ0 IOC4*$_{W22A\ K149E\ K150E}$*-3xFlag::HIS3*	This study

Table S1 Yeast strains used in this study

**Data collection**	
Space group	P63
Cell dimension	
a,b,c(Å)	153.303, 153.303, 42.004
α,β,γ(°)	90.000, 90.000, 120.000,
Resolution (Å)	50.0-2.30(2.34 -2.30) *
R_{meg} (%)	10.9/(70.6)
$I/\sigma I$	35.5(5.6)
Completeness (%)	99.9(100.0)
Redundancy	7.9(7.8)
**Refinement**	
Resolution (Å)	2.3
Total No. reflection / free	25341/1995
R_{work} / R_{free}	0.173/0.229
r.m.s.d. bonds/angles (Å)	0.008/1.117
Protein/solvent atoms	3910/ 300
Average B-factors(Å^2)	35.451
**Ramachandran plot statistics**	
Most favorable	98.57%
Additionally allowed	1.22%
Disallowed	0.20%

Table S2 Statistics of crystallographic analysis

*Values in parentheses are for highest-resolution shell.

Primer name	Primer sequence
**PCR primers**	
147-601-for (Cy5)	5'- GGGTCTAGACAATACATGCACAGGATGTA -3'
147-601-rev	5'- TCTAGAGTCGGGAGCTCGGA -3'
147-601-for (IRD700)	5'- GGGTCTAGACAATACATGCACAGGATGTA -3'
147-601-rev	5'- TCTAGAGTCGGGAGCTCGGA -3'
147-601-for (IRD800)	5'- GGGTCTAGACAATACATGCACAGGATGTA -3'
147-601-rev	5'- TCTAGAGTCGGGAGCTCGGA -3'
215-601-for (Cy5)	5'- GGGTCTAGAGGCAAGGTCGCTGTTCAATA -3'
215-601-rev (mid)	5'- GGGGGATCCTATGTGATGGACCCTATACG -3'
215-601-for (IRD700)	5'- GGGTCTAGAGGCAAGGTCGCTGTTCAATA -3'
215-601-rev (mid)	5'- GGGGGATCCTATGTGATGGACCCTATACG -3'
215-601-for (IRD800)	5'- GGGTCTAGAGGCAAGGTCGCTGTTCAATA -3'
215-601-rev (mid)	5'- GGGGGATCCTATGTGATGGACCCTATACG -3'
**EMSA probes**	
$EMSA_{sense}$ (Cy5)	5'- GGC AAG GTC GCT GTT CAA TAC ATG CAC AGG -3'
$EMSA_{antisense}$	5'- CCT GTG CAT GTA TTG AAC AGC GAC CTT GCC -3'
$EMSA_{sense}$ AT rich (Cy5)	5'- GGC AAG GUC GCU GUU CAA UAC AUG CAC AGG -3'
$EMSA_{antisense}$ AT rich	5'- CCU GUG CAU GUA UUG AAC AGC GAC CUU GCC -3'
$EMSA_{sense}$ GC rich (Cy5)	5'- CCC GGU GCC GAG GCC GCU CAA UUG G -3'
$EMSA_{antisense}$ GC rich	5'- CCA AUU GAG CGG CCU CGG CAC CGG G -3'
**RT-qPCR primers**	
ACT1_F	5'-CAAGGTATCATGGTCGGTATGG-3'
ACT1_R	5'-CGTGTTCAATTGGGTAACGTAAAG-3'
ARO80_F	5'-GTGATCTTGGTCCGGTTGAT-3'
ARO80_R	5'-AGTCGTTCGAAGTTCCCTTATT-3'
FAA2_F	5'-TCTTGTGGTGCCATAGGTATTT-3'
FAA2_R	5'-CCACGAATTTGCAGTTCACC-3'
VTH2_F	5'-TAGGAGACGATGGGCTGATAG-3'
VTH2_R	5'-GAAACGCAGACTTGGTGATTAAAG-3'
YEN1_F	5'-TATCGGAAAGGTAGGCGAGT-3'
YEN1_R	5'-GAGCCGGTATTTCTTCCAGAC-3'
**ChIP-qPCR primers**	
ADE13_F	5'-CCTCCAGACAAGAAGTACATGAG-3'
ADE13_R	5'-CCACCTTCTTCCTTGACTACTG-3'
CHD1-pr_F	5'-CACATACTTGTTTCTTCCAAAGGAG-3'
CHD1-pr_R	5'-AATAGGTAAGCTAGTGGAACTCAAG-3'
CHD1-mid_F	5'-AATCACAGGCACACCTCTTC-3'
CHD1-mid_R	5'-CGTAAACCTTCCGGGCATTA-3'
PCA1-pr_F	5'-TGAACGAATGTATGGGTTAGGT-3'
PCA1-pr_R	5'-GCACCTTATTTATGCCAAGTGTAG-3'
PCA1-mid_F	5'-CTTACAGGCCTCTTCTGCTATTC-3'
PCA1-mid_R	5'-CTGTTCCATCAGTTGGGATTCT-3'
PYK1_F	5'-TCAACGCCGTTACCACTATG-3'
PYK1_R	5'-AGGTTGGCTTTGGAGTACAG-3'
VAS1_F	5'-TTATCGTGCGTCCAGATTAGTT-3'
VAS1_R	5'-CATCATAGCCTGGGACTGATAAA-3'
ChrV_F	5'-GGCTGTCAGAATATGGGGCCGTAGTA-3'
ChrV_R	5'-CACCCCGAAGCTGCTTTCACAATAC-3'
STE3_F	5'-GTTCGCAAGGCTGTTGATATTC-3'
STE3_R	5'-GTGTCCTTCTACCTGCTGTAAA-3'

Table S3 Primers used in this study

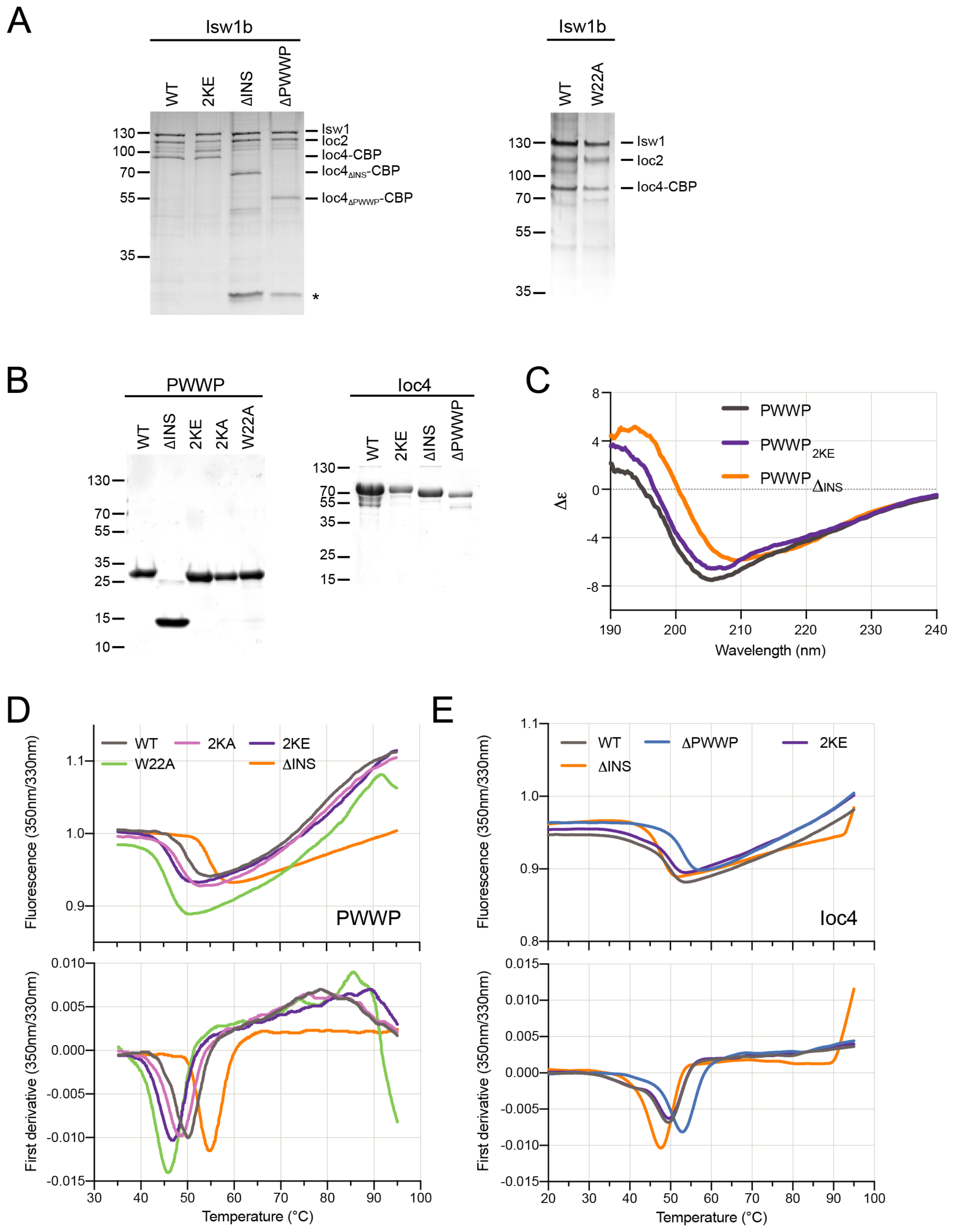

Figure S1 Protein purification and stability. **(A)** Silver stained SDS-PAGE of TAP-purified wildtype and mutant Isw1b remodeler complexes. TEV protease was identified by mass spectrometry and is indicated (*). **(B)** SDS-PAGE analysis of purified, wildtype and mutant PWWP and Ioc4 constructs. Proteins were stained with Coomassie Blue. **(C)** Circular dichroism analysis of pufified PWWP constructs shows that all proteins are folded. **(D,E)** Nano differential scanning fluorimetry (DSF) results for purified PWWP **(D)** and Ioc4 **(E)** proteins. The PWWP constructs were analysed using the Nanotemper Tycho, the Ioc4 proteins were run on the Nanotemper Prometheus. Results are not directly comparable due to different settings for the temperature ramp.

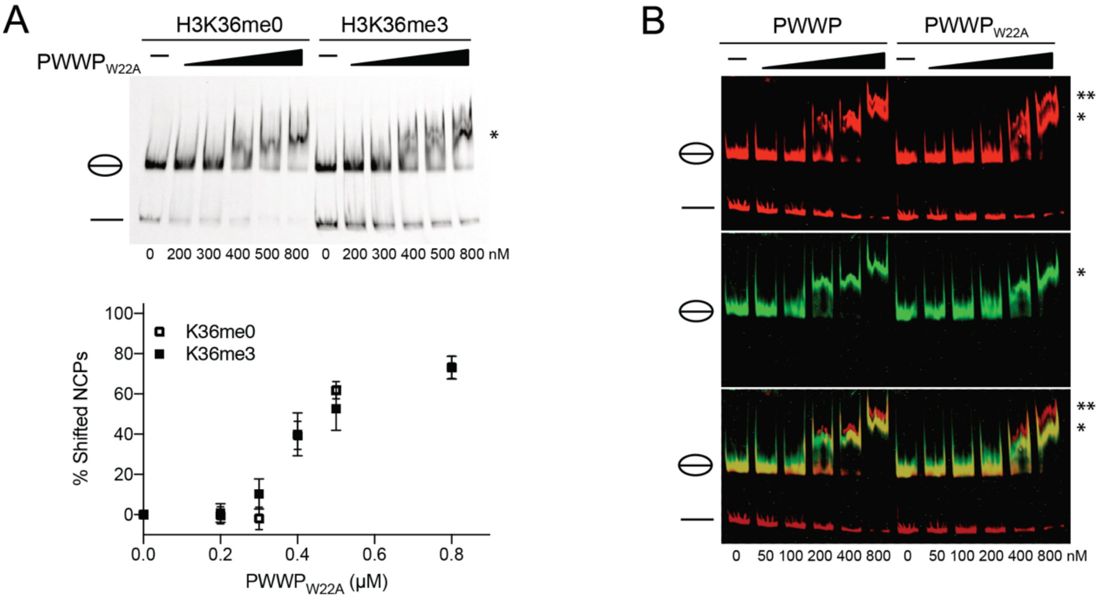

Figure S2 The PWWP aromatic cage is important for H3K36me3 recognition. EMSAs **(A)** and competitive EMSAs **(B)** were performed to assess the ability of the aromatic cage mutant W22A to specifically recognise H3K36 trimethylated nucleosomes. As expected, mutation of W22 led to a loss of discrimination in both settings. Furthermore, the mutant PWWP domain displayed lower affinities towards nucleosomes in general, when compared to the wildtype domain. The positions of the NCP and free DNA bands on the gels are indicated, as are the bands denoting the complexes formed by the PWWP proteins with NCPs (*) as well as with free DNA (**).

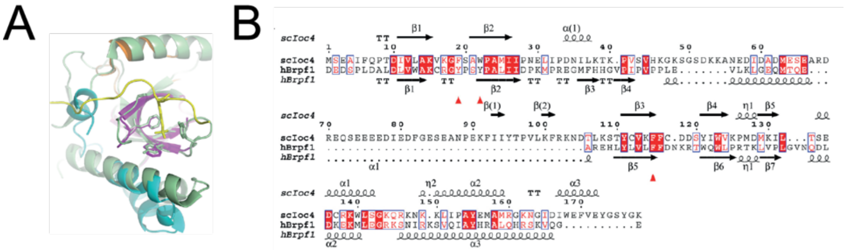

Figure S3 Structural and sequence conservation of PWWP domains **(A)** Overlay of the PWWP domains from Ioc4 (colored as in Fig. 1H) and hBrpf1 (light green). The H3K36me3-containing peptide is shown in yellow. Residues forming the aromatic cage are shown as sticks. **(B)** Sequence alignment of PWWP domains from Ioc4 (scIoc4) and hBrpf1. The alignment was generated with Clustal Omega and displayed with ESPript (http://espript.ibcp.fr/). Secondary structure elements of the Ioc4 and Brpf1 PWWP domains are marked at the top and bottom of the alignment, respectively. Identical residues are highlighted in red. Aromatic cage residues accommodating the histone trimethyllysine are labeled with red triangles.

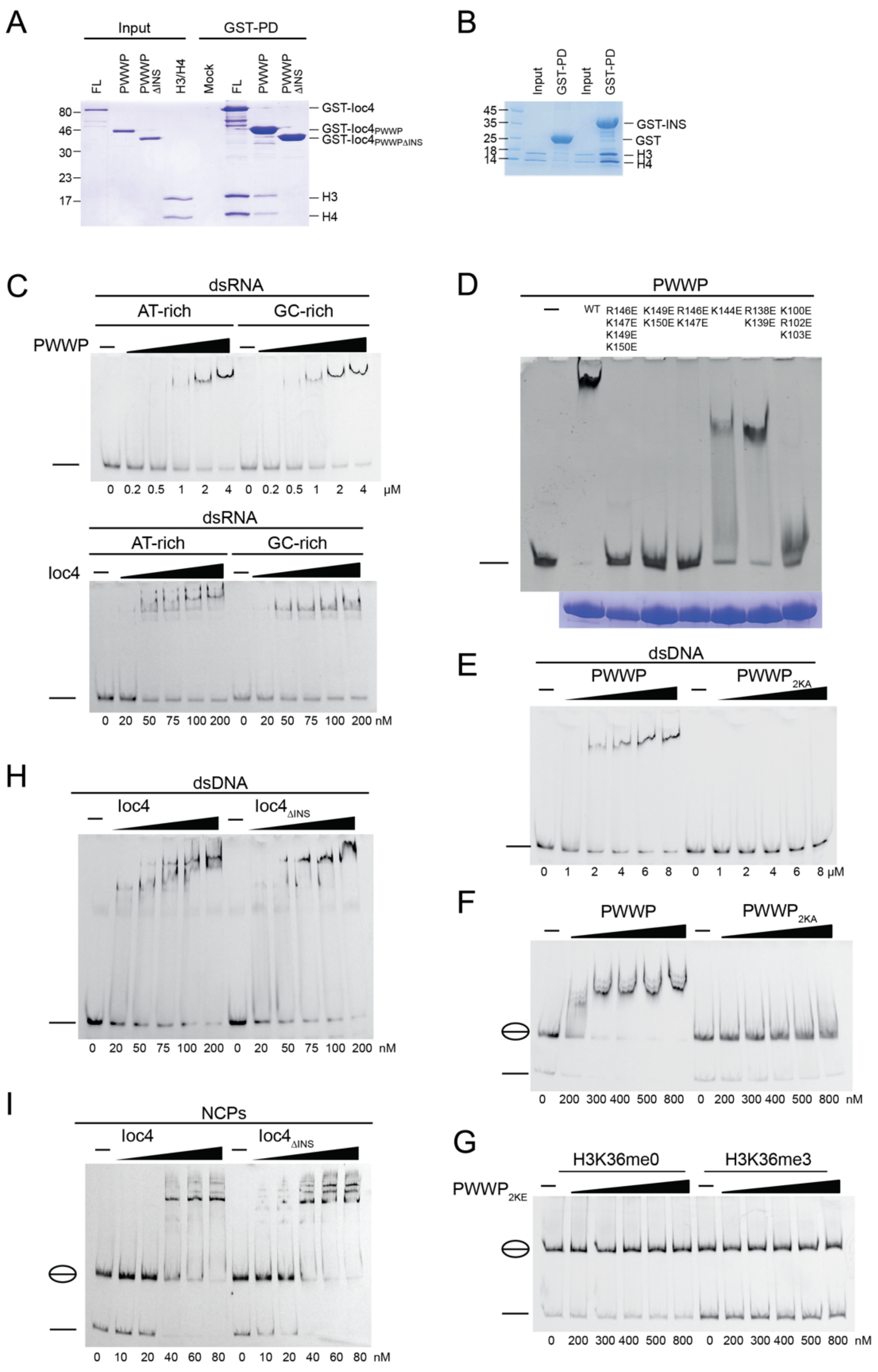

Figure S4 Interaction of Ioc4 and Ioc4$_{PWWP}$ constructs with histones and nucleosomes **(A)** Pull-down assays of GST-tagged Ioc4, PWWP and PWWP without its insertion motif (PWWP$_{\Delta INS}$) with histone H3/H4 tetramers. **(B)** Pull-down assays of GST-tagged insertion motif (INS) with histone H3/H4 tetramers. **(C)** EMSA of wildtype PWWP and Ioc4 with dsRNA. **(D)** Wildtype and mutant PWWP domains were purified, used to set up binding reactions with double-stranded DNA (30 bp) and analyzed by EMSA. The DNA bands are indicated by a line. Equal protein loading is shown. **(E)** EMSA of wildtype PWWP and PWWP$_{2KA}$ with dsDNA (30 bp). **(F)** EMSA of wildtype PWWP and PWWP$_{2KA}$ with unmodified NCPs. **(G)** EMSA of PWWP$_{2KE}$ with unmethylated and trimethylated H3K36 NCPs. **(H)** EMSA of Ioc4 and Ioc4$_{\Delta INS}$ with dsDNA. **(I)** EMSA of Ioc4 and Ioc4$_{\Delta INS}$ with NCPs. Free NCPs and/or DNA are indicated.

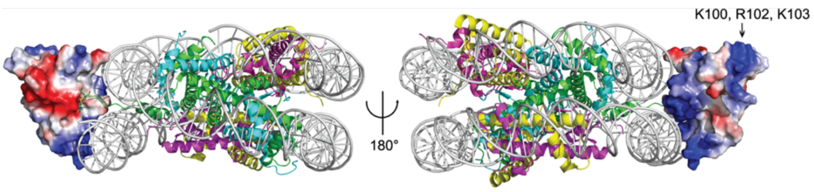

Figure S5 Model of Ioc4 PWWP binding to NCP. Homology Model based on Wang et al. (2). The electrostatic surface of the Ioc4-PWWP is shown. Basic residues (K100, R102, K103) part of the insertion motif are indicated.

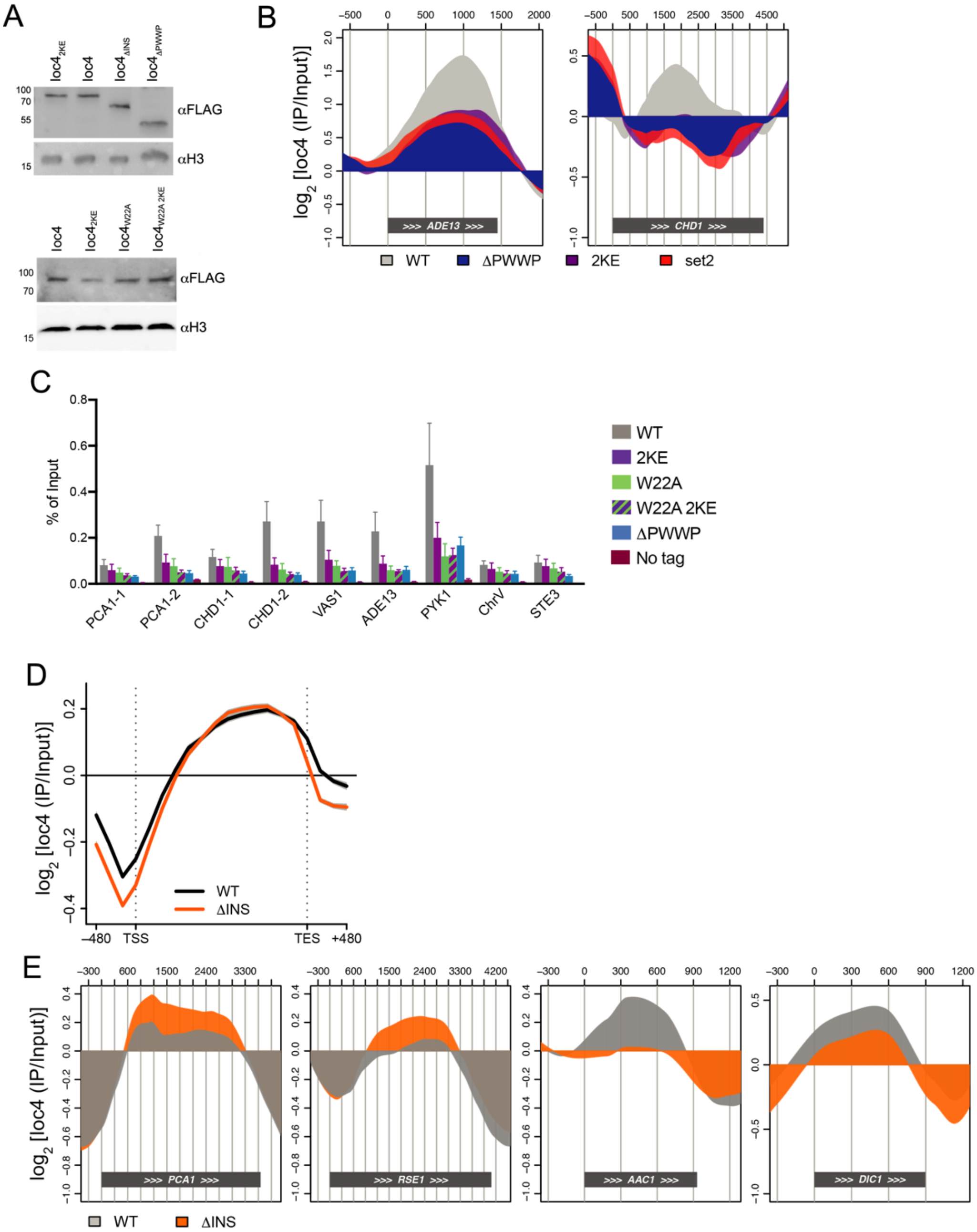

Figure S6 Ioc4 recruitment to chromatin. **(A)** Western blot of whole cell extracts from yeast strains expressing wildtype or mutant 3xFlag tagged Ioc4. Histone H3 was used as a loading control. **(B)** Localization of wildtype and mutant Ioc4 over individual genes. (**C**) ChIP-qPCR experiments were performed for wildtype and mutant IOC4-3xFlag strains. BY4741 was used as an untagged control. Localization was determined over promoter and ORF regions. *STE3* and *ChrV* served as control regions. All ChIP signals were normalized to input. **(D)** Metagene analysis of ChIP-chip experiments using yeast genome tiling arrays. Whole-genome average data (n=6451 genes) for three independent experiment were plotted as mean ± s.e.m. (gray) for wildtype Ioc4 and Ioc4$_{\Delta INS}$. (**E)** Localization of wildtype Ioc4 and Ioc4$_{\Delta INS}$ over individual genes.

Supplementary methods

Yeast strains and media

All yeast strains used in this study are listed in Table S2. Wildtype *IOC4* was tagged with a 3xFLAG epitope by targeted homologous integration of a PCR product derived from amplification of plasmid p3xFLAG-HIS3 with gene-specific primers. In order to generate yeast strains bearing different mutations of *IOC4*, mutant *IOC4* constructs were first cloned into plasmid p3xFLAG-HIS3, followed by PCR amplification of these cassettes and transformation into wildtype yeast. To generate TAP-tagged yeast strains, the 3xFLAG tag was replaced by targeted homologous integration of a PCR product derived from amplification of plasmid pBS1539 with construct-specific primers. Single deletion of *CHD1* was done by targeted homologous recombination of PCR fragments containing either the hygromycin (*HphB*) or kanamycin (*KanMX*) resistance marker. All strains generated in this study were confirmed by PCR and/or sequencing. Cells were grown at 30°C in YPD (1% yeast extract, 2% bacto-peptone, 2% dextrose) medium.

Yeast growth assay

Yeast strains were inocculated at an OD_{600} of 0.1 from overnight cultures and grown for ca. 5 hours at 30°C in YPD until they were growing exponentially. Equal numbers of cells were harvested by centrifugation, washed with ddH_2O and resuspended at an OD_{600} of 0.5 in ddH_2O. Five 6-fold dilutions were prepared for each strain and spotted onto YPD plates +/- 1μM propiconazole. Plates were incubated at 30°C for 3-5 days.

Protein purification

For crystallization the Ioc4 PWWP domain (aa 1-178; $Ioc4_{PWWP}$) was cloned into a modified pMAL-c2X vector (New England Biolabs, Liu *et al.*, 2001) in order to produce a fusion protein with an N-terminal maltose-binding protein (MBP). Overexpression of MBP-$Ioc4_{PWWP}$ in *Escherichia coli* BL21 (DE3) was induced by the addition of 0.25 mM IPTG for 20 hours at 16 °C. MBP-$Ioc4_{PWWP}$ was purified to homogeneity using a sequence of amylose resin affinity chromatography, anion exchange and gel filtration chromatography. The PWWP domain of wildtype (aa 1-178) and the $PWWP_{2KE}$ mutant were cloned into a pRSF vector with an N-terminal 6xHis-tag. Both proteins were overexpressed in *E. coli* BL21(DE3) by the addition of 0.25 mM IPTG for 20 hours at 16 °C. Both proteins were purified to homogeneity using nickel-NTA resin, followed by anion exchange chromatography. Full-length Ioc4, PWWP and $PWWP_{\Delta INS}$ were cloned into pGEX6T1 and overexpressed in *E. coli* BL21(DE3). GST-PWWP and GST-$PWWP_{\Delta INS}$ cultures were induced by addition of 0.25 mM IPTG for 20 hours at 16ºC. Full-length GST-Ioc4 was produced as described previously (1). All GST-tagged proteins were purified using glutathione-sepharose as described previously (3). If needed, GST tags were removed by cleavage with 3C protease while bound to glutathione-sepharose. Proteins were dialyzed exhaustively against 50 mM phosphate, pH 7.0, 500 mM NaCl, 10% Glycerol, flash-frozen in liquid nitrogen and stored at -80 °C. Full-length Ioc4 (aa1-475), $Ioc4_{2KE}$ and $Ioc4_{\Delta PWWP}$ (aa 179-475) were cloned into a modified pCoofy vector with an N-terminal 6xHis-MBP tag. Proteins were overexpressed in *E. coli* BL21 RIL by addition of 0.25 mM IPTG for 20 hours at 16 °C. Proteins were batch purified using nickel-NTA resin, followed by adsorption chromatography using a heparin column and gel filtration chromatography. Proteins were dialyzed exhaustively against 50 mM phosphate, pH 8.0, 500 mM NaCl, 10% Glycerol, 50 mM Arg, 50 mM Glu, flash-frozen in liquid nitrogen and stored at -80 °C.

TAP-tagged wildtype and mutant Isw1b chromatin remodelers were purified from *Saccharomyces cerevisiae* as described previously. Briefly, yeast were grown in YPD at 30°C until they reached an OD_{600} of 5-7. Cells were harvested by centrifugation at 5,000 x *g* for 15 min and washed twice with cold PBS. Cells were resuspended in 20 ml of TAP Extraction Buffer (40 mM HEPES-KOH, pH7.5, 350 mM NaCl, 10% Glycerol, 0.1% Tween-20, 1 mM PMSF, 2 μg/ml Leupeptin, 1 μg/ml Pepstatin A) and lysed using a freezer mill. Lysates were treated with 100 μl of 10 mg/ml Heparin and 10 μl of Benzonase (25 U/μl, Merck Millipore) for 15 minutes at room temperature before removing cell debris by centrifugation at 31,000 x *g* for 20 minutes and ultracentrifugation at 208,000 x *g* for 1.5 hours. The supernatant was incubated with pre-washed IgG sepharose (GE Healthcare) and incubated at 4 °C for 3 hours. The resin was washed with TAP extraction buffer for three times 5 min, resuspended with TEV cleavage buffer (10 mM Tris pH 8.0, 10% Glycerol, 150 mM NaCl, 0.1% IGEPAL CA630, 0.5 mM EDTA, 1 mM DTT) and incubated with TEV protease for 16 hours at 4°C. Cleaved protein was collected and applied to

calmodulin sepharose (GE Healthcare) pre-washed with binding buffer (10 mM Tris, pH8.0, 150 mM KCl, 1 mM Magnesium acetate, 1 mM Imidazole, 2 mM $CaCl_2$, 10% Glycerol, 0.1% IGEPAL CA630, 1 mM DTT) for 3 hours at 4°C. The resin wash washed with binding buffer for three times 10 min. The purified complexes were eluted with elution buffer (10 mM Tris, pH8.0, 150 mM KCl, 1 mM Magnesium acetate, 1 mM Imidazole, 10 mM EGTA, 10% Glycerol, 0.1% IGEPAL CA630, 0.5 mM DTT), concentrated and subsequently flash frozen in liquid nitrogen and stored at -80°C.

Recombinant *Xenopus laevis* histones were expressed, purified and assembled into core histone octamers as described (3). To generate histone H3 containing either unmethylated or trimethylated K36, tail peptides (aa1-44) containing either K36me0 or K36me3 were ligated onto histone $H3_{T45C}$ and used for subsequent core histone octamer assembly (4). For slinding assays methyl-lysine analogue (MLA) K36C nucleosomes chemically modified to resemble trimethylated H3K36 were used instead, as described previously (1).

Crystallization and structure determination

Purified MBP-$Ioc4_{PWWP}$ was concentrated to approximately 10 mg/ml and used for crystal screening by sitting-drop vapor diffusion method at 16°C. Needle-shaped crystals grew in reservoir solution containing 0.1 M Potassium thiocyanate, 30% Polyethylene glycol monomethyl ether 2,000. Data were collected on beamline BL17U (SSRF, China, λ = 0.979 Å) and processed using the HKL2000 program suite. Structure of the fusion protein was solved by molecular replace method with the program PHASER, using the MBP structure as a search template. An initial model was automatically built by Phenix Autosol, manually modified with Coot and refined with Phenix Refine. The final model contains two missing fragments, F19 and S43-K91 and has an R_{work} of 17.3% and an R_{free} of 22.9%. Data scaling, refinement, and validation statistics are shown in Table S1.

GST pull-down assays

GST-tagged Ioc4 constructs were incubated with 2 µg of recombinant, reconstituted histone H3/H4 tetramers and 10 µl of washed glutathione sepharose in buffer B (20 mM Tris-HCl, pH 7.5, 200 mM KCl, 0.1% NP-40, 20% Glycerol, 0.2 mM EDTA, 1 mM PMSF, 1 µg/ml pepstatin A, 2 µg/ml leupeptin) for 2.5 hours at 4 °C. Beads were washed with 3x 1 ml of buffer B containing 300 mM KCl, eluted with SDS-PAGE loading buffer and analyzed by SDS-PAGE on 15% gels.

Reconstitution of recombinant mononucleosomes

DNA fragments measuring either 147 bp or 215 bp and containing the 601 positioning sequence were PCR amplified from pGEM-3Z/601 (5) using a 5' Cy5-labeled and a 3' primer (Table S3). Mononucleosomes were reconstituted from DNA and recombinant *c*ore histone octamers by serial dilution as described (3,6).

Electromobility shift assays (EMSA)

Binding of $Ioc4_{PWWP}$ to DNA was assessed by EMSA. Double-stranded (ds) DNA with a length of 30 bp was prepared by heating and annealing complimentary, single-stranded (ss) DNA (Table S3). Gels were stained with ethidium bromide and visualized using a Tanon 1600 gel imaging system (Tanon Inc.). Alternatively, DNA was labeled with Cy5 and visualized by scanning on a Typhoon FLA9500 Imaging system and quantitated using ImageQuant TL software (GE Healthcare). DNA bands were quantitated for each lane. All lanes containing PWWP were normalized against input lanes containing DNA only. The percentage of DNA bound was expressed as 100 - % DNA for each lane. Mean values ± SEM were plotted for at least three independent experiments.

EMSA assays with reconstituted, K36me0- or K36me3-containing mononucleosomes were performed as described (3). For binding reactions 15 fmol of reconstituted nucleosomes per reaction were incubated with increasing concentrations (0-800 nM) of wildtype and mutant PWWP domains. For binding reactions with full-length Ioc4 15 fmol of reconstituted nucleosomes per reaction were incubated with increasing concentrations (0-35 nM) of wildtype or mutant Ioc4. For binding reactions with unmodified wildtype nucleosomes 30 fmol of reconstituted nucleosomes per reaction were incubated with increasing concentrations (0-4 µM) of wildtype or mutant PWWP. Complexes were separated by electrophoresis on 5.0 % native polyacrylamide gels (37.5:1), run in 0.4x TBE, 2 % glycerol. Gels were scanned using a Typhoon Imaging FLA9500 system and quantitated using ImageQuant TL software (GE Healthcare).

Mononucleosome bands were quantitated for each lane. All lanes containing PWWP were normalized against input lanes containing mononucleosomes only. The percentage of nucleosomes bound was expressed as 100 - % mononucleosomes for each lane. Mean values ± SEM were plotted for at least three independent experiments.

Remodelling assays
For nucleosome sliding assays, 10 µl reactions were set up, containing 30 fmol mononucleosomes and 10 fmol Isw1b complex in buffer R (50 mM Tris-HCl, pH8.0, 50 mM KCl, 10 mM $MgCl_2$, 1 mM ATP, 0.1 µg/µl BSA, 0.5 mM PMSF). Reactions were incubated at room temperature and aliquots removed at various time points and stopped by adding 720 ng plasmid DNA, 500 mM KCl. Nucleosomes were resolved by electrophoresis on 7 % native polyacrylamide gels (37.5:1) in 0.4x TBE, 2 % glycerol. Gels were scanned using a Typhoon FLA9500 Imaging system and quantitated using ImageQuant TL software (GE Healthcare). Bands representing remodelled mononucleosomes were normalized to their respective input lanes.

Antibodies
The following antibody was used in this study: αFlag M2 (Sigma #F1804).

Chromatin immunoprecipitation assays
For ChIPs of FLAG-tagged Ioc4 yeast strains were grown in 200 ml of YPD at 30 ºC, crosslinked and processed for ChIP as described earlier (7,8). FLAG-tagged Ioc4 was immunoprecipitated and processed as described before (7,8). Three biological repeats were done for all experiments and used for subsequent ChIP-qPCR and/or ChIP-chip analyses.

ChIP-qPCR analysis
Immunoprecipitated DNA was quantitated by qPCR using the PowerTrack SYBR Green Master Mix (Thermo Fisher Scientific) and a QuantStudio 5 Real-Time PCR System (Applied Biosystems). Primers are listed in Table S3. The mean signal was calculated for each experiment and normalized against input samples at each primer positions as internal controls. Input-normalized values were further corrected for variation by normalising against the mean signals for two control regions (*STE3,* subtelomeric region on chromosome V (*ChrV*)).

ChIP-chip microarray analysis
ChIP-chip assays for the genome-wide distribution of FLAG-tagged Ioc4 were performed as described previously (1), using 8x60K yeast genome DNA arrays (Agilent, Array #031697) with an average probe spacing of ca. 200 bp. 20-50 ng of input and IP samples were used for double T7 linear amplification and labeling. Inputs were labeled with Cy3 dye and IPs with Cy5 dye. 4 µg of input and IP were combined and used for hybridization. Arrays were scanned (Agilent DNA Microarray Scanner Model G2505B) and extracted using Feature Extraction software (Agilent) and normalized using median normalization in R software.

Data analysis
The normalized data were analyzed using a modified gene average analysis (1). ORFs were subdivided into 14 equal sised bins each. Intergenic regions (480 bp up- and downstream of genes) were allocated into three bins each. Microarray enrichment ratios [log_2(IP/Input)] for each probe were assigned to the closest bin. For whole-genome average gene plots all probes within a bin were averaged and plotted as mean ± standard error (SEM). Genes that are not regulated by RNAPII, including tRNA and snRNA genes as well as the majority of dubious ORFs (ca. 450 genes) were removed from the analysis.

Isolation of total RNA
Yeast strains were grown in YPD at 30ºC until they reached an OD_{600} of 0.8. Total RNA was isolated using acid phenol extraction as described previously (9). RNA quality and quantity were assessed by UV spectroscopy using a NanoDrop 2000 (Thermo Scientific).

Northern blots
Northern blotting and hybridization were done as described previously (10). 20 µg of total RNA were used to assess the cryptic transcript phenotype. Blots were exposed onto phosphorimaging screens and scanned using a Typhoon FLA9500 Imaging system.

Strand-specific multiplex RT-qPCR

Total RNA was treated with TURBO DNA-free DNase I (ThermoScientific) to remove genomic DNA according to the manufacturer's instructions. 3.5 µg of DNase-treated RNA were used for subsequent reverse transcription (RT) reactions containing 2 pmol of each gene-specific primer, 60 U of SuperScript III (ThermoScientific) and 0.3 µg of Actinomycin D (AppliChem) to prevent antisense cDNA artefacts. Two RT reactions containing "forward" primers (Table S3) annealing to antisense transcripts derived from target genes *VTH2* and *YEN1,* or *FAA2* and *ARO80* were set up. Each reaction also contained 2 pmol "reverse" primer annealing to the canonical reference *ACT1* transcript. Annealing was done at 70°C for 10 minutes. First strand synthesis was performed at 55°C for one hour, followed by heat inactivation at 70°C for 15 min. All samples were quantitated by qPCR using the PowerTrack SYBR Green Master Mix (Thermo Fisher Scientific) and a QuantStudio 5 Real-Time PCR System (Applied Biosystems). Ct-values were first normalized to those of *ACT1.* Subsequently, results for mutant yeast strains were normalized to the mean signal for wildtype yeast samples using the comparative CT (ΔΔCT) method. Five biological replicates were performed for each strain.

References

1. Smolle, M., Venkatesh, S., Gogol, M.M., Li, H., Zhang, Y., Florens, L., Washburn, M.P. and Workman, J.L. (2012) Chromatin remodelers Isw1 and Chd1 maintain chromatin structure during transcription by preventing histone exchange. *Nat Struct Mol Biol*, **19**, 884-892.
2. Wang, H., Farnung, L., Dienemann, C. and Cramer, P. (2020) Structure of H3K36-methylated nucleosome-PWWP complex reveals multivalent cross-gyre binding. *Nat Struct Mol Biol*, **27**, 8-13.
3. Li, B., Gogol, M., Carey, M., Lee, D., Seidel, C. and Workman, J.L. (2007) Combined action of PHD and chromo domains directs the Rpd3S HDAC to transcribed chromatin. *Science*, **316**, 1050-1054.
4. Voigt, P., LeRoy, G., Drury, W.J., 3rd, Zee, B.M., Son, J., Beck, D.B., Young, N.L., Garcia, B.A. and Reinberg, D. (2012) Asymmetrically modified nucleosomes. *Cell*, **151**, 181-193.
5. Thastrom, A., Bingham, L.M. and Widom, J. (2004) Nucleosomal locations of dominant DNA sequence motifs for histone-DNA interactions and nucleosome positioning. *J Mol Biol*, **338**, 695-709.
6. Li, B., Jackson, J., Simon, M.D., Fleharty, B., Gogol, M., Seidel, C., Workman, J.L. and Shilatifard, A. (2009) Histone H3 lysine 36 di-methylation (H3K36ME2) is sufficient to recruit the Rpd3S histone deacetylase complex and to repress spurious transcription. *J Biol Chem*, **284**, 7970-7976.
7. Strahl-Bolsinger, S., Hecht, A., Luo, K. and Grunstein, M. (1997) SIR2 and SIR4 interactions differ in core and extended telomeric heterochromatin in yeast. *Genes Dev*, **11**, 83-93.
8. Li, B. and Reese, J.C. (2001) Ssn6-Tup1 regulates RNR3 by positioning nucleosomes and affecting the chromatin structure at the upstream repression sequence. *J Biol Chem*, **276**, 33788-33797.
9. Li, B., Gogol, M., Carey, M., Pattenden, S.G., Seidel, C. and Workman, J.L. (2007) Infrequently transcribed long genes depend on the Set2/Rpd3S pathway for accurate transcription. *Genes Dev*, **21**, 1422-1430.
10. Carrozza, M.J., Li, B., Florens, L., Suganuma, T., Swanson, S.K., Lee, K.K., Shia, W.J., Anderson, S., Yates, J., Washburn, M.P. *et al.* (2005) Histone H3 methylation by Set2 directs deacetylation of coding regions by Rpd3S to suppress spurious intragenic transcription. *Cell*, **123**, 581-592.
